# The effect of propolis addition to the laying-hen diet on performance, serum lipid profile and liver fat rate

**DOI:** 10.5194/aab-66-225-2023

**Published:** 2023-09-06

**Authors:** Şaziye Canan Bölükbaşı, Hilal Ürüşan, Betül Apaydın Yıldırım

**Affiliations:** 1 Department of Animal Science, Faculty of Agriculture, Atatürk University, Erzurum, Türkiye; 2 Plant and Animal Production Department, Technical Sciences Vocational School, Erzurum, Türkiye; 3 Department of Biochemistry, Faculty of Veterinary, Atatürk University, Erzurum, Türkiye

## Abstract

The aim of this study was to evaluate the effect of propolis (P) on performance, egg quality parameters, serum lipid profile, some liver enzymes and liver fat ratio. One-hundred-and-twenty Lohmann (LSL) laying hens were divided into five groups, and each group consisted of six subgroups. The control group was fed basal diet. The other groups were fed high-energy (HE) diets to induce fatty liver syndrome, and 0, 100, 200 and 300 mg kg ${}^{-1}$ of propolis were supplemented with high-energy feeds. During the 8-week trial, feed and water were given ad libitum.

It was determined that egg production and feed conversion ratio were decreased in the high-energy feed group without the addition of propolis. The highest egg production was found in HE + 100 and HE + 200 mg kg ${}^{-1}$ of P groups. It was found that liver fat ratios were higher in the group fed with HE + 0 mg kg ${}^{-1}$ of P feed ( P<0.01 ) than other groups. But the addition of P decreased the liver fat rate significantly. The highest very low density lipoprotein (VLDL), triglyceride (TG) and low-density lipoprotein (LDL) values were found for the HE + 0 mg kg ${}^{-1}$ of P group. The addition of 200 mg kg ${}^{-1}$ of P to high-energy feed increased glutathione (GSH), superoxide dismutase (SOD), catalase (CAT) and glutathione peroxidase (GPx) values.

In conclusion, high-energy feed adversely affected egg production and liver fat ratio, but the addition of 100 or 200 mg kg ${}^{-1}$ of propolis improved egg production and decreased liver fat ratio.

## 1 Introduction

In order to meet egg requirements, which have an important place in human nutrition, the caged layer hen system is widely practiced today. In caged layer hens, the amount of product obtained is high because more animals are housed per unit area. However, this situation causes some problems such as fatty liver syndrome. For laying hens raised in cages, fat can occur in the liver due to the restriction in the movement area and the high-energy value of the feeds (Shini et al., 2019). “Fatty liver”, which is called hepatosteatosis in medical language, means excessive accumulation of fat in liver cells. Fatty liver is a disease characterized by an accumulation of fat in the abdominal cavity and a decrease in egg production. The disease causes a shrinkage of egg numbers in laying hens, a drop of 45 % in egg production within a few days and subsequent deaths (Crespo et al., 2013). It was reported that the amount of liver fat is significantly affected by dietary fat (Butler, 1976).

This disease, which is associated with feeding birds with a high-energy feed, is most common during the summer period. The liver is usually enlarged, pale and fragile. Although there is no known method for the treatment of fatty liver syndrome, its development can be prevented by the use of lipotropic agents such as vitamin E, vitamin B12 and choline chloride.

Propolis (P) is a resinous substance collected by bees from leaves, stems and buds of various plants, mixed with bee enzymes, pollen and wax. Propolis is composed of many components, primarily polyphenol components, such as phenolic acids, esters, phenolic aldehydes and ketones. The antiseptic, antibacterial, anti-inflammatory, immunomodulatory, antioxidant, antimutagenic and cytotoxic effects of propolis have been proven in scientific studies. In addition, its protective effect on liver has been reported by many researchers (Bhadauria et al., 2009; Kısmet et al., 2008; Paulino et al., 2014; Wali et al., 2015; Omar et al., 2016). Most of the biological effects of propolis are related to its antioxidant capacity.

To the best of our knowledge, there is no study on the effect of propolis on fatty liver in laying hens. In this study, in order to see the effect of propolis more clearly, we aimed to examine the effect of propolis supplementation on performance, liver fat ratio, egg quality and some antioxidant enzymes by promoting fatty liver in layer hens fed high-energy feed.

## 2 Materials and methods

### 2.1 Experimental animals and experimental design

The study was conducted in accordance with the ethics committee principles of Atatürk University veterinary faculty (2018/1). The aim of this study was to evaluate the effect of propolis on performance and fatty liver in laying hens. Laying hens were fed with a high-energy feed (2850 kcal kg ${}^{-1}$ ) to induce fatty liver. One-hundred-and-twenty white Lohmann (LSL) laying hens at an age of 70 weeks were used. The hens were divided into five groups, and each group consisted of six subgroups. The birds were placed in four-story battery cages ( 60×59×61 cm) with six hens in each as replicates. The first group was the control group where the birds were fed with basal feed (Table 1), while the other groups were fed with feeds that added 0, 100, 200 and 300 mg kg ${}^{-1}$ of propolis (P) to high-energy feeds. During the 8-week trial, feed and water were given ad libitum. The propolis used in the diet was obtained from a commercial company.

**Table 1:** Composition of feeds used in the trial (%).

Item	Basal diet	High-energy
	(control)	diet
Corn (8.5 % CP)	63	64.67
Soybean meal (44 % CP–46 % CP)	16.39	13.50
Corn gluten (60 % CP)	8.48	10.64
Limestone	9.68	7.65
DCP 18	1.44	1.44
Soybean oil	0.17	1.68
Vitamin–mineral mixture ${}^{*}$	0.25	0.25
Salt	0.22	0.33
Sodium bicarbonate	0.16	0.16
L-Lysine	0.11	0.10
DL-Methionine 99 %	0.10	0.08
Calculated composition (%)		
Dry matter	88.41	88.24
Crude protein	17.52	17.48
Ether extract	2.20	3.84
Crude ash	11.87	10.95
Crude fiber	2.78	2.97
D-Methionine	0.38	0.38
Methionine	0.40	0.41
Lysine	0.76	0.70
ME kcal kg ${}^{-1}$	2726	2850
Analyzed composition (%)		
Dry matter	88.78	88.00
Crude protein	17.12	17. 34
Ether extract	2.43	3.63
Crude ash	11.24	11.45
Crude fiber	3.18	3.29

CP: crude protein. ME: metabolic energy. DCP: dicalcium phosphate. IU: international unit. ${}^{*}$ per kilogram diet added: 12 000 IU vitamin A; 2500 IU vitamin D3; 30 IU vitamin E; 4 mg vitamin K3; 3 mg vitamin B1; 6 mg vitamin B2; 30 mg niacin; 10 mg calcium D-pantothenate; 5 mg vitamin B6; 0.015 mg vitamin B12; 1 mg folic acid; 0.050 mg D-biotin; 50 mg vitamin C; 300 mg choline chloride; 80 mg manganese; 60 mg iron; 60 mg zinc; 5 mg copper; 0.5 mg cobalt; 0.2 mg iodine; 0.15 mg selenium.

### 2.2 Performance analysis

Feed consumption, egg production, egg weight and feed conversion ratio (kg feed kg ${}^{-1}$ egg) values of the birds were determined with the measurements made every 2 weeks. Likewise, egg quality criteria such as shell thickness, breaking strength, albumen ratio, yolk ratio, shell ratio, shape index and Haugh unit were determined by the measurements made every 2 weeks.

### 2.3 Blood parameter analysis

At the end of the experiment, blood samples that were taken from the vein of one animal from each subgroup ( n=6 ) and placed into heparinized tubes were centrifuged (3000 rpm for 10 min), and the samples were stored at - 80 ${}^{\circ }$ C.

Superoxide dismutase (SOD) activity (Sun et al., 1988), glutathione (GSH) level (Tietze, 1969), MDA (malondialdehyde) level (Yoshioka et al., 1979), glutathione peroxidase (GPx) activity (Matkovics et al., 1988), catalase (CAT) activity (Goth, 1991), TP (total protein) levels (Lowry, 1951) and non-esterified fatty acid (NEFA) levels in plasma (Biont Chicken NEFA ELISA Kit, cat. no. YLA0179CH) were measured with a BioTek ELISA reader (BioTek μ Quant MQX200 ELISA reader, USA). TP levels were used to calculate the SOD and GPx activity. Plasma cholesterol, glucose, LDL (low-density lipoprotein), HDL (high-density lipoprotein), VLDL (very low density lipoprotein), AST (aspartate transaminase), ALT (alanine transaminase) and TG (triglyceride) values were analyzed in a special laboratory.

At the end of the experiment, one animal from each subgroup was slaughtered. Liver wet weights were determined ( n=6 ); then they were brought to the laboratory and dried at 105 ${}^{\circ }$ C where their dry weights were determined. Then, the fat ratio was determined by ether extraction in the dried samples (Kutlu, 2008).

### 2.4 Statistical analyses

For performance values, egg quality criteria, some blood parameters and antioxidant enzyme values, variance analyses were performed by the “general linear model” procedure, and the importance controls of the important data were performed using the SPSS 17 program. Differences between groups were found by Duncan's multiple comparison test.

## 3 Results

The values of the feed consumption, egg weight, egg production and feed conversion ratio of the experimental groups are given in Table 2.

No significant difference was found between the control group, the HE + 0 mg kg ${}^{-1}$ of P group and the HE + 300 mg kg ${}^{-1}$ of P group in terms of feed intake. However, it was determined that adding 100 and 200 mg kg ${}^{-1}$ of P to feed increased feed intake significantly ( P<0.05 ). There was no significant difference between groups in terms of egg weight. It was determined that egg production was significantly lower in HE + 0 and HE + 300 mg kg ${}^{-1}$ of P groups compared to the control group, but the addition of 100 and 200 mg kg ${}^{-1}$ of P to a high-energy diet significantly increased egg production ( P<0.01 ). In the HE + 0 mg kg ${}^{-1}$ of P and HE + 300 mg kg ${}^{-1}$ of P groups, the degree of feed conversion ratio decreased significantly ( P<0.05 ).

**Table 2:** The effect of propolis on the performance of laying hens consuming high-energy feed.

Groups	Feed	Egg	Egg	Feed
	consumption	weight	production	conversion
	(g)	(g)	(%)	ratio (g : g)
Control	115.01b	61.58	79.97b	2.35b
HE + 0 mg kg ${}^{-1}$ of P	110.26b	61.46	71.51c	2.62a
HE + 100 mg kg ${}^{-1}$ of P	127.16a	61.67	87.19a	2.38b
HE + 200 mg kg ${}^{-1}$ of P	128.88a	63.29	86.33a	2.39b
HE + 300 mg kg ${}^{-1}$ of P	109.44b	60.53	68.57c	2.77a
SE	2.61	0.35	1.95	0.06
P	${}^{*}$	ns	${}^{**}$	${}^{*}$

a–c: the averages shown with different letters in the same column are different from each other. HE: high energy. P: propolis. Control: basal-fed group. HE + 0 mg kg ${}^{-1}$ of P: high-energy-fed group + 0 mg kg ${}^{-1}$ of propolis. HE + 100 mg kg ${}^{-1}$ of P: high-energy-fed group + 100 mg kg ${}^{-1}$ of propolis. HE + 200 mg kg ${}^{-1}$ of P: high-energy-fed group + 200 mg kg ${}^{-1}$ of propolis. HE + 300 mg kg ${}^{-1}$ of P: high-energy-fed group + 300 mg kg ${}^{-1}$ of propolis. SE: standard error. ns: not significant. ${}^{*}$ P<0.05 . ${}^{**}$ P<0.01 .

There was no significant difference between the groups in terms of values of egg quality criteria such as albumen ratio, yolk ratio, shell ratio, Haugh unit, shell breaking strength or shell thickness (Table 3).

**Table 3:** The effect of propolis on egg quality of laying hens consuming high-energy feed.

Groups	Albumen	Yolk	Egg shell	Hough	Shell	Shell
	(%)	(%)	(%)	unit	breaking	thickness
					strength	(mm)
					(kg cm ${}^{2}$ )	
Control	61.25	28.66	10.09	80.21	2.40	0.449
HE + 0 mg kg ${}^{-1}$ of P	57.45	31.91	10.63	83.55	2.11	0.472
HE + 100 mg kg ${}^{-1}$ of P	56.57	32.23	11.19	83.58	3.21	0.481
HE + 200 mg kg ${}^{-1}$ of P	58.16	31.37	10.46	85.75	3.15	0.431
HE + 300 mg kg ${}^{-1}$ of P	58.45	29.97	11.61	81.03	2.91	0.482
SE	0.60	0.50	0.20	0.90	0.26	0.008
P	ns	ns	ns	ns	ns	ns

a–c: the averages shown with different letters in the same column are different from each other. HE: high energy. P: propolis. Control: basal-fed group. HE + 0 mg kg ${}^{-1}$ of P: high-energy-fed group + 0 mg kg ${}^{-1}$ of propolis. HE + 100 mg kg ${}^{-1}$ of P: high-energy-fed group + 100 mg kg ${}^{-1}$ of propolis. HE + 200 mg kg ${}^{-1}$ of P: high-energy-fed group + 200 mg kg ${}^{-1}$ of propolis. HE + 300 mg kg ${}^{-1}$ of P: high-energy-fed group + 300 mg kg ${}^{-1}$ of propolis. SE: standard error. ns: not significant.

The means of wet weight, dry weight and fat ratio of liver are given in Table 4. The lowest values in terms of wet and dry liver weights were determined in the control and HE + 300 mg kg ${}^{-1}$ of P groups. The difference among the experimental groups in terms of liver fat ratio was significant ( P<0.01 ); the highest value was found in the HE + 0 mg kg ${}^{-1}$ of P group, while the lowest value was observed in the HE + 300 mg kg ${}^{-1}$ of P group. Liver fat ratios of the HE + 100 and HE + 200 mg kg ${}^{-1}$ of P groups were found to be considerably lower than the HE + 0 mg kg ${}^{-1}$ of P group.

**Table 4:** The effect of propolis on wet weight (g), dry weight (g) and fat ratio (%) of liver of laying hens consuming high-energy feed.

Groups	Wet weight	Dry weight	Fat ratio of
	of liver (g)	of liver (g)	liver % (DM)
Control	21.34b	8.16b	25.52c
HE + 0 mg kg ${}^{-1}$ of P	32.75a	13.00a	56.66a
HE + 100 mg kg ${}^{-1}$ of P	35.02a	10.74a	32.00b
HE + 200 mg kg ${}^{-1}$ of P	31.03a	11.26a	28.50b
HE + 300 mg kg ${}^{-1}$ of P	25.50b	8.74b	21.00d
SE	1.84	0.82	3.65
P	${}^{*}$	${}^{*}$	${}^{**}$

a–c: the averages shown with different letters in the same column are different from each other. DM: dry matter. HE: high energy. P: propolis. Control: basal-fed group. HE + 0 mg kg ${}^{-1}$ of P: high-energy-fed group + 0 mg kg ${}^{-1}$ of propolis. HE + 100 mg kg ${}^{-1}$ of P: high-energy-fed group + 100 mg kg ${}^{-1}$ of propolis. HE + 200 mg kg ${}^{-1}$ of P: high-energy-fed group + 200 mg kg ${}^{-1}$ of propolis. HE + 300 mg kg ${}^{-1}$ of P: high-energy-fed group + 300 mg kg ${}^{-1}$ of propolis. SE: standard error. ns: not significant. ${}^{*}$ P<0.05 . ${}^{**}$ P<0.01 .

There was no significant difference between the groups in terms of ALT, AST, glucose, total cholesterol and HDL cholesterol. The difference between the groups in terms of VLDL, TG and LDL cholesterol was significant ( P<0.01 ), with the highest values seen in the HE + 0 mg kg ${}^{-1}$ of P group. It was observed that the NEFA level increased significantly in the HE + 300 mg kg ${}^{-1}$ of P group.

**Table 5:** The effect of propolis on some blood plasma biochemistry parameters of laying hens consuming high-energy feed.

	Control	HE + 0 mg kg ${}^{-1}$ of P	HE + 100 mg kg ${}^{-1}$ of P	HE + 200 mg kg ${}^{-1}$ of P	HE + 300 mg kg ${}^{-1}$ of P	SEM	P
VLDL (mg dL ${}^{-1}$ )	96.00b	304.50a	157.33b	65.66b	113.66b	26.36	0.020
ALT U (L ${}^{-1}$ )	3.00b	7.00b	3.00	3.66	3.66	0.66	ns
AST U (L ${}^{-1}$ )	256.6	314.50	218.33	313.33	265	18.92	ns
Glucose (mg dL ${}^{-1}$ )	257.00	245.50	259.33	288.00	277.66	6.64	ns
Total cholesterol (mg dL ${}^{-1}$ )	130.9	124.00	70.33	138.00	102.00	11.33	ns
TG (mg dL ${}^{-1}$ )	419.60b	1323.00a	785.00b	328.66b	568.00b	131.87	${}^{*}$
HDL (mg dL ${}^{-1}$ )	43.00	35.5	27	49.66	30.66	4.96	ns
LDL (mg dL ${}^{-1}$ )	100.20b	198.00a	113.33b	48.66b	55.00b	17.52	${}^{**}$
NEFA (ng L ${}^{-1}$ )	0.219b	0.220b	0.221b	0.211b	0.505a	0.035	${}^{**}$

a–c: the averages shown with different letters in the same row in the are different from each other. HE: high energy. P: propolis. Control: basal-fed group. HE + 0 mg kg ${}^{-1}$ of P: high-energy-fed group + 0 mg kg ${}^{-1}$ of propolis. HE + 100 mg kg ${}^{-1}$ of P: high-energy-fed group + 100 mg kg ${}^{-1}$ of propolis. HE + 200 mg kg ${}^{-1}$ of P: high-energy-fed group + 200 mg kg ${}^{-1}$ of propolis. HE + 300 mg kg ${}^{-1}$ of P: high-energy-fed group + 300 mg kg ${}^{-1}$ of propolis. SE: standard error. ns: not significant. ${}^{*}$ P<0.05 . ${}^{**}$ P<0.01 .

There was a significant difference ( P<0.01 ) between the groups in terms of MDA, GSH, SOD, CAT and GPx values (Table 6). The highest plasma MDA value and the lowest GSH, SOD, CAT and GPx values were detected in the HE + 300 mg kg ${}^{-1}$ of P group.

Contrary to expectations, the MDA value in this study was determined to be the highest in the HE + 300 mg kg ${}^{-1}$ of P group, not in the HE + 0 % P group. It was observed that MDA levels increased significantly in parallel with the NEFA value in the HE + 300 mg kg ${}^{-1}$ of P group (Table 5).

**Table 6:** The effect of propolis on MDA and some enzyme activity of liver of laying hens consuming high-energy feed.

Groups	MDA	GSH	SOD	CAT	GPx
	(nmol L ${}^{-1}$ )	(nmol L ${}^{-1}$ )	(U L ${}^{-1}$ )	(U L ${}^{-1}$ )	(U L ${}^{-1}$ )
Control	7.64b	2.30b	58.16ab	147.29b	1.47b
HE + 0 mg kg ${}^{-1}$ of P	7.34b	2.49a	58.12ab	152.63ab	1.47b
HE + 100 mg kg ${}^{-1}$ of P	7.84b	2.15c	55.94b	152.83ab	1.46b
HE + 200 mg kg ${}^{-1}$ of P	7.55b	2.57a	60.12a	162.97a	1.57a
HE + 300 mg kg ${}^{-1}$ of P	20.39a	1.61d	50.47c	110.09c	1.20c
SEM	1.55	0.10	1.08	5.86	0.038
P	${}^{**}$	${}^{**}$	${}^{**}$	${}^{**}$	${}^{**}$

a–d: the averages shown with different letters in the same column are different from each other. HE: high energy. P: propolis. Control: basal-fed group. HE + 0 mg kg ${}^{-1}$ of P: high-energy-fed group + 0 mg kg ${}^{-1}$ of propolis. HE + 100 mg kg ${}^{-1}$ of P: high-energy-fed group + 100 mg kg ${}^{-1}$ of propolis. HE + 200 mg kg ${}^{-1}$ of P: high-energy-fed group + 200 mg kg ${}^{-1}$ of propolis. HE + 300 mg kg ${}^{-1}$ of P: high-energy-fed group + 300 mg kg ${}^{-1}$ of propolis. SE: standard error. ns: not significant. ${}^{**}$ P<0.01 .

## 4 Discussion

It has been reported that propolis has antioxidant, antimutagenic and immunomodulatory properties, and these properties are due to its rich flavonoid, phenolic acid and terpenoid content (Prytzyk et al., 2003; Wang et al., 2003). The antioxidant properties of propolis are thought to be the cause of the increase in egg production and feed conversion ratio. In addition, the increase in feed consumption has been attributed to the aroma and anti-lipidemic effect of propolis. Similarly to this study, Galal et al. (2008) reported that the addition of propolis to laying-hen rations increased feed consumption, egg production, egg weight and improved feed efficiency. El-Neney and Awadien (2016) reported that layer hens fed diets supplemented with different levels of propolis (0.1, 0.2 or 0.3 g kg ${}^{-1}$ ) significantly improved feed efficiency per hen, egg production, egg weight and egg mass. It has been reported that the addition of 1 g kg ${}^{-1}$ of P in Japanese quails (Denli et al., 2005) and 2 g kg ${}^{-1}$ of P in ducks improved feed conversion ratio (Abdel-Rahman and Mosaad, 2013). Abdel-Kareem and El-Sheikh (2017) found that adding different levels of propolis to laying-hen feed did not affect feed intake but increased egg production and feed conversion ratio. Contrary to these studies, Belloni et al. (2015) reported that as the level of dietary propolis content (1.0 % to 3.0 %) in the layer's diet increased, feed intake decreased. They suggested that this decrease in feed consumption may be due to the aroma of propolis. Navarro-Villa et al. (2019) reported that egg production decreased significantly in laying hens fed with high-energy and low-protein feed to promote liver formation.

In this study, no effect of propolis on egg quality criteria was detected. Similarly, it has been reported that the addition of propolis to laying-hen rations does not affect the albumen, yolk or egg shell ratio but does increase the Hough unit and shell thickness (Galal et al., 2008). Özkök et al. (2013) reported that propolis doses (0.1, 0.2 or 0.4 g kg ${}^{-1}$ ) had no dietary effect on quality criteria such as Haugh units or shell thickness. However, Vilela et al. (2012) reported that propolis has a positive effect on the internal content of egg and quality of the egg shell. Abdel-Kareem and El-Sheikh (2017) reported that the addition of 1000 mg kg ${}^{-1}$ of P to laying-hen diets increased the weight of yolk and shell and the Haugh unit but did not affect albumen weight.

It was determined that the addition of propolis to the diet increased the wet and dry weights of the liver in this study. Similarly, Hassan and Abdulla (2011) found that the addition of 400 mg kg ${}^{-1}$ of P to broiler diets increased liver weight.

It has been reported that the rate of liver fat exceeds 40 % of the dry weight and can even reach 70 % in the case of fatty liver (Ivy and Nesheim, 1973). In the present study, it was determined that the liver fat ratio (56.66 %) was higher in the group fed with high-energy feed (HE + 0 % P) compared to the other groups. Zhuang et al. (2019) observed that the rate of liver fat increased significantly in their study in which they fed laying hens with a high-energy low-protein diet to induce fatty liver hemorrhagic syndrome. Similarly, in many studies conducted in previous years, it was reported that the rate of liver fat increased in animals fed with high-energy feed (Splittgerber et al., 1969; Jensen et al., 1970; Akkılıç and Tanyolaç, 1975). Rozenboim et al. (2016) reported that in laying hens fed a high-fat diet, the liver fat rate in young animals was not affected by diet, but the liver fat rate in old animals was lower than in the control group. Unlike mammals, fat synthesis is high in the liver of birds (Hermier, 1997). The liver plays a major role in fat synthesis and metabolism in laying hens. Similar to mammals, in avian species the digestion and absorption of dietary fat occurs in the small intestine (Tancharoenrat et al., 2014). However, due to the poorly developed intestinal lymphatic system in birds, dietary fatty acids are discharged directly into the portal blood system (instead of the lymphatic system) in the form of very low density lipoproteins (VLDL) called “portomicrons” (Bensadoun and Rothfeld, 1972). Bird livers become more prone to fat accumulation, as most portomicrons travel from the portal blood system to the liver before reaching the rest of the circulation (Cherian et al., 2002).

Similar to this study, Navarro-Villa et al. (2019) reported that liver fat ratio increased significantly in laying hens fed with high-energy and low-protein feed to promote fatty liver formation. In the current study, it was determined that adding propolis to the high-energy diet significantly reduced the liver fat ratio compared to the control group. Lin et al. (1997) reported that 30 mg kg ${}^{-1}$ of P can prevent fatty liver degeneration caused by prolonged alcohol intake in humans.

Studies on the properties of propolis have shown that its addition to the diet protects liver tissue against the negative effects of various hepatotoxic factors (Banskota et al., 2000; Bazo et al., 2002; Bhadauria et al., 2009).

Unlike this study, in a study in which different levels of propolis were added to laying-hen diets, it was reported that a level of 1000 mg kg ${}^{-1}$ of P increased total protein level and decreased ALT, AST and cholesterol levels (Abdel-Kareem and El-Sheikh, 2017). Galal et al. (2008) reported that blood triglyceride, cholesterol and ALT levels were significantly reduced in laying hens consuming 100–150 mg kg ${}^{-1}$ of P. Zhuang et al. (2019) found that the amount of ALT increased significantly in layer hens fed with a high-energy low-protein diet compared to the control group. In a study by Attia et al. (2014), it was reported that blood triglyceride and cholesterol concentrations were significantly reduced in chickens supplemented with 300 mg kg ${}^{-1}$ of P continuously for 35 d. The researchers argued that the decrease in these values was beneficial and safe by minimizing the liver function and reducing the harmful effects on the tissues by propolis treatment.

Koya-Miyata et al. (2009) found that propolis (5 mg kg ${}^{-1}$ for 10 d) significantly reduced triglyceride, cholesterol, and NEFA levels in high-fat-diet mice. Hashem et al. (2013) reported in a study they conducted on rabbits that the addition of 150 mg kg ${}^{-1}$ of P reduced cholesterol and triglyceride levels; it had no effect on LDL cholesterol and increased HDL cholesterol. In a study conducted in patients with non-alcoholic fatty liver disease, it was reported that the addition of propolis to the diet reduced the level of total cholesterol (Nikbaf-Shandiz et al., 2022). Kısmet et al. (2017) reported in their study on mice with non-alcoholic fatty liver syndrome that the addition of 100 and 200 mg of propolis to the ration reduced total cholesterol, triglyceride, HDL cholesterol, ALT and AST levels in serum.

In fatty liver symptom, overload of the non-esterified fatty acid (NEFA) level has been reported to trigger reactive oxidative stress formation through mitochondria-dependent oxidation or microsomal enzymes (Kısmet et al. 2017). Contrary to this study, Hashem et al. (2013) reported that the addition of 150 mg kg ${}^{-1}$ of P reduced MDA level in rabbits.

Catalase (CAT), glutathione peroxidase (GPx) and superoxide dismutase (SOD) are involved in the enzymatic antioxidant defense system of the body (Arya et al., 2021). While the highest SOD, CAT and GPx values were found in the 200 mg kg ${}^{-1}$ of P group, the lowest values were found in the 300 mg kg ${}^{-1}$ of P group in this study. And the lowest GSH value was determined in the 300 mg kg ${}^{-1}$ of P group.

On the other hand, Arya et al. (2021) reported in a study they conducted on patients with non-alcoholic fatty liver syndrome that SOD and GPx levels were lower than the control group, and the MDA value was higher. Similar to this study, it has been reported that there is a negative correlation between MDA and SOD in some other studies (Koruk et al., 2004; Videla et al., 2004).

## 5 Conclusions

As a result, it was determined that the liver fat ratio increased significantly and egg production decreased in laying hens fed with a high-energy feed. However, it has been determined that the addition of 200 mg kg ${}^{-1}$ of propolis to the high-energy diet increases egg production; significantly reduces the liver fat rate; decreases the amounts of LDL and triglycerides; and increases SOD, CAT, and GPx activities. It has been determined that especially 200 mg kg ${}^{-1}$ of propolis may be used in laying-hen rations since it has a positive effect on many parameters, but it has also been concluded that more studies are needed on the subject.

## Data Availability

The data presented in this study are available free of charge for any user upon reasonable request from the corresponding author.
